# Synchronized Activity in The Main and Accessory Olfactory Bulbs and Vomeronasal Amygdala Elicited by Chemical Signals in Freely Behaving Mice

**DOI:** 10.1038/s41598-017-10089-4

**Published:** 2017-08-30

**Authors:** Cecília Pardo-Bellver, Sergio Martínez-Bellver, Fernando Martínez-García, Enrique Lanuza, Vicent Teruel-Martí

**Affiliations:** 1 Department of de Biologia Cellular, Facultat de Ciències Biològiques, Universitat de València, Burjassot, Spain; 2 Laboratori de Circuits Neurals, Department of d’Anatomia i Embriologia Humana, Facultat de Medicina, Universitat de València, Valencia, Spain; 3 Unitat Predepartamental de Medicina, Facultat de Ciències de la Salut, Universitat Jaume I. Castelló de la Plana, Castelló, Spain

## Abstract

Chemosensory processing in mammals involves the olfactory and vomeronasal systems, but how the activity of both circuits is integrated is unknown. In our study, we recorded the electrophysiological activity in the olfactory bulbs and the vomeronasal amygdala in freely behaving mice exploring a battery of neutral and conspecific stimuli. The exploration of stimuli, including a neutral stimulus, induced synchronic activity in the olfactory bulbs characterized by a dominant theta rhythmicity, with specific theta-gamma coupling, distinguishing between vomeronasal and olfactory structures. The correlated activation of the bulbs suggests a coupling between the stimuli internalization in the nasal cavity and the vomeronasal pumping. In the amygdala, male stimuli are preferentially processed in the medial nucleus, whereas female cues induced a differential response in the posteromedial cortical amygdala. Thus, particular theta-gamma patterns in the olfactory network modulates the integration of chemosensory information in the amygdala, allowing the selection of an appropriate behaviour.

## Introduction

Most terrestrial vertebrates possess two major olfactory systems. The main olfactory system detects and processes all kind of volatile chemicals in the environment^1^, whereas the accessory olfactory (or vomeronasal) system is devoted mainly to the detection and processing of volatile and involatile chemicals with biological relevance^2^, e.g. chemosignals. Such chemicals include sexual pheromones^3, 4^, chemical signals eliciting aggressive behaviour^5, 6^, predator cues^7^, illness-derived cues^8^, and stress-related signals^9^. Therefore, the main olfactory system allows the animal to analyse its chemical environment, whereas the vomeronasal system is mainly involved in the detection of conspecifics and predators. Anatomically, the projections of the main and accessory olfactory bulbs are largely segregated^10^. The main olfactory bulb (MOB) innervates primarily the anterior olfactory nucleus, piriform cortex and some cortical amygdaloid nuclei. In contrast, the accessory olfactory bulb (AOB) mainly projects to the medial (Me) and posteromedial cortical (PMCo) amygdaloid nuclei, and the posterior bed nucleus of the *stria terminalis* ^11^. However, although olfactory and vomeronasal pathways reach different structures, information from both the olfactory and vomeronasal systems should be integrated to allow the generation of a complete picture of the chemicals present in the environment^12, 13^. Noteworthy, there is a relatively minor, but relevant, direct convergence of olfactory and vomeronasal information in some amygdaloid structures, particularly in the anterior Me^10, 14–17^.

From the electrophysiological perspective, the olfactory system shows a prominent oscillatory activity, apparently coupled to the respiratory rhythm^18^. This oscillatory profile includes predominant waves in the theta (4–12 Hz), beta (12–30 Hz) and gamma (30–100 Hz) frequency bands. This rhythmical activity is involved in the discrimination of odorants^19^ and in olfactory learning^20^. However, little is known about the oscillatory activity in the vomeronasal system, with some information available for the AOB^21, 22^, and the Me^22^, in this latter case in the context of a social recognition paradigm.

The vomeronasal organ, where the receptor neurons are located, presents a particular mechanism to introduce high molecular weight molecules into the organ^23, 24^ named vomeronasal pumping. Hypothetically, repeated contraction and dilatation of a blood vessel located in the lumen of the organ generates successive negative and positive pressure able to suck stimuli into the organ and clear the lumen in a cyclic manner. In contrast, in the main olfactory system oscillatory activity is strongly dependent on the respiratory rhythm, and on active sniffing behaviour coupled to this rhythm^25^. It is unknown whether the mechanisms of sniffing and vomeronasal pumping are independent and, consequently, their cyclic activities generate different and independent patterns of oscillations in the main and accessory olfactory systems. Alternatively, olfactory sniffing and vomeronasal pumping could work in a synchronic fashion and, accordingly, activity in the vomeronasal system would be coupled to the rhythmical activity described in the main olfactory system.

To investigate the oscillatory pattern of activity in the vomeronasal system, and compare it with that showed by the olfactory system, we have performed recordings of the Local Field Potentials (LFP) in awake, freely behaving mice to which we presented olfactory stimuli (clean bedding or geraniol), or mixed olfactory-vomeronasal stimuli (bedding soiled by females, castrated males or intact males). In each animal, the recording electrodes were located in the MOB and AOB, as well as in the Me and PMCo. These recording sites allow us to characterize the pattern of oscillatory activity in the main centres of the vomeronasal system, and at the same time, to evaluate whether they are different and independent from the sniffing-induced olfactory oscillations present in the MOB.

## Results

**Figure 1:**
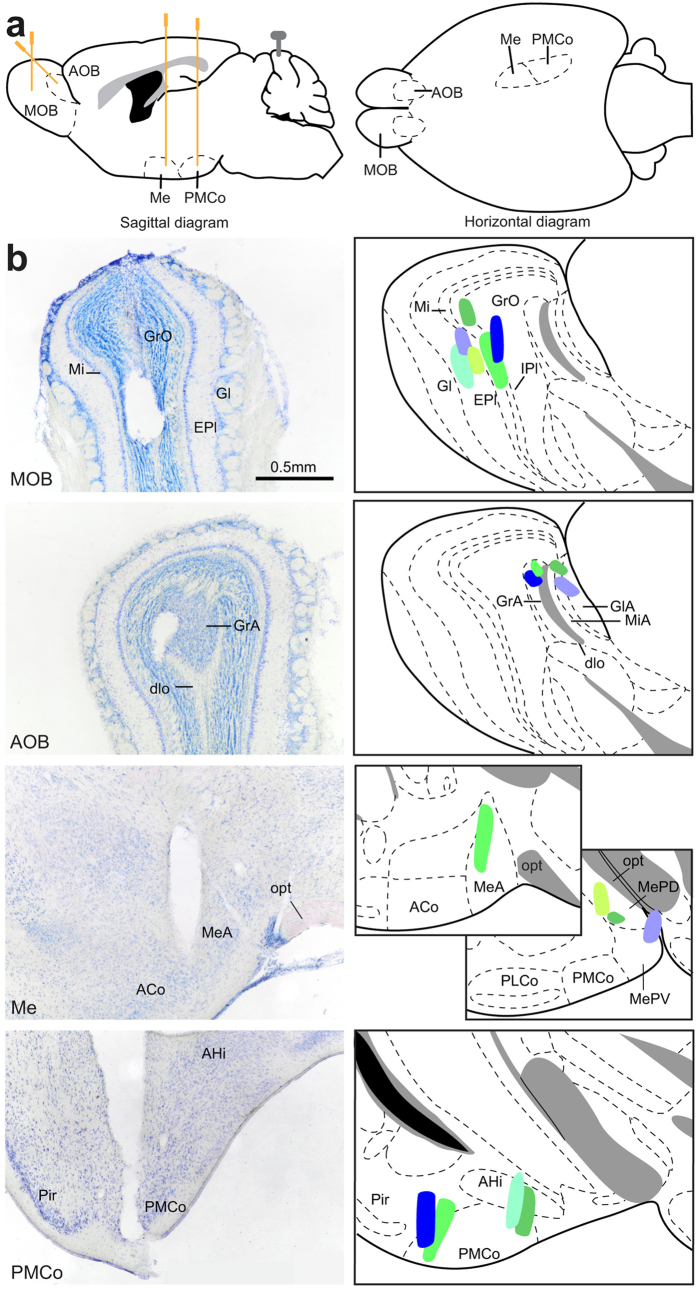
(**a**) Schematic diagram of the recording sites, sagittal diagram (left) and horizontal diagram (right). (**b**) Verification of the electrodes position. The actual recording site (electrode tip) should be along the trace of the electrode. Right, histological verification of the four electrodes placed on the same animal. Left, schematic drawings of the recording regions on all the mice; each colour indicates a different animal. Abbreviations: ACo, anterior cortical amygdaloid nucleus; AHi, amygdalo-hippocampal transition zone; AOB, accessory olfactory nucleus; dlo, olfactory tract, dorsal part; EPl, external plexiform layer of the MOB; Gl, glomerular layer of the MOB; GlA, glomerular layer of the AOB; GrA, granular layer of the AOB; GrO, granular layer of the MOB; IPl, internal plexiform layer of the MOB; Me, medial amygdaloid nucleus; MeA, medial amygdaloid nucleus, anterior subdivision; MePD, medial amygdaloid nucleus, posterodorsal subdivision; MePV, medial amygdaloid nucleus, posteroventral subdivision; Mi, mitral layer of the MOB; MiA, mitral layer of the AOB; MOB, main olfactory bulb; *opt*, optic tract; PLCo, posterolateral cortical amygdaloid nucleus; PMCo, posterimedial cortical amygdaloid nucleus.

Electrophysiological recordings were carried out in the brain of freely behaving adult female mice under an olfactory exploration paradigm. The animal was placed in a clean cage and the stimuli were presented in a restricted area by means of a Petri dish containing clean, aromatised, or soiled bedding. After recording, only those cases in which the electrode tip was clearly positioned within the boundaries of the target areas were included for the study (n = 6; Fig. 1).

Attending to our experimental paradigm, we first analysed the exploratory behaviour induced by the different olfactory stimuli. The analysis of the time spent investigating the different stimuli revealed significant differences (*F* = 5.24, p < 0.0004; Supplementary Fig. 1). Post-hoc comparisons (Tukey test) revealed three homogeneous subsets of means (*p* < 0.05): clean and geraniol-scented bedding, *p* = 0.995 (neutral stimuli), geraniol-scented bedding and female-soiled bedding (*p* = 0.093), and female, castrated male and male-soiled bedding (*p* = 0.993). Therefore, the investigation time elicited by the conspecific stimuli was higher than that induced by neutral stimuli, although female-soiled bedding was not different from geraniol-scented bedding.

### Neuronal activity under exploratory behaviour

In general, the study of the neural activity in the different nodes of the bulb-amygdaloid network showed a similar profile, where the exploration periods could clearly be distinguished from those in which a passive behaviour was prevailing. We first analysed the population activity under the presence of different stimuli evoking olfactory activation in the AOB.

**Figure 2:**
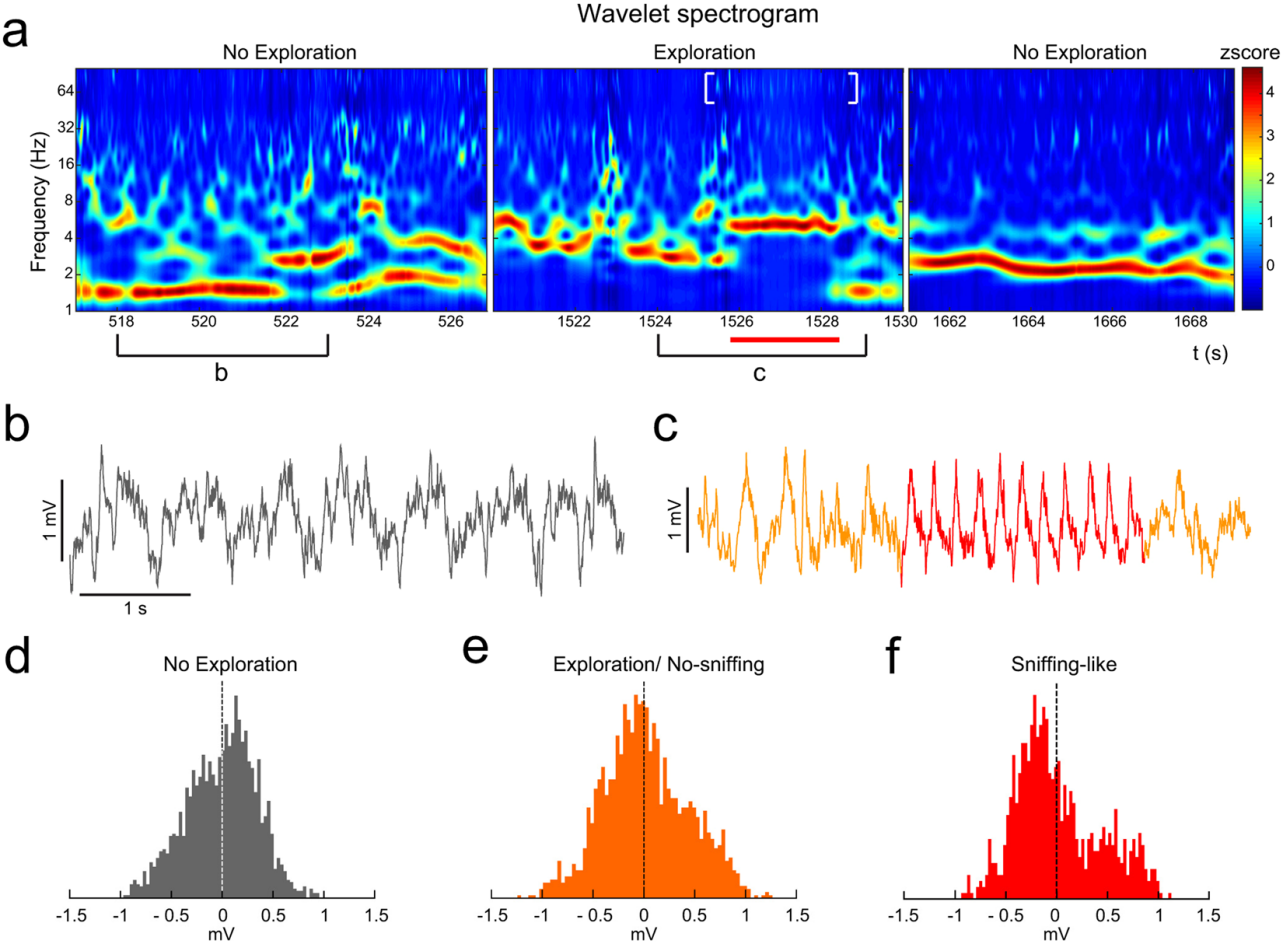
Oscillatory changes induced by clean soiled bedding presentation. (**a)** Wavelet spectrogram of three different time periods in the AOB; left panel: non-exploration time; middle panel: exploration of a stimulus (red line indicating a sniffing-like oscillation and white brackets indicating gamma activity); right panel: non-exploration after the stimulus. Colour bar indicates the wavelet power coefficients (z-scored). (**b)** Raw trace during non-exploration time. (**c)** Raw trace during exploration time (in red indicating a *sniffing*-like oscillation). (**d–f)** Distribution of the amplitudes, with differential asymmetry of the waveform. (**d)** In a non-exploration time. (**e)** In an exploration/no-sniffing time. (**f)** In a sniffing-like oscillation present under exploratory behaviour.

The LFP showed distinguishable profiles in the absence and presence of stimuli, easily delimited in the time-frequency domain by the wavelet spectrograms. This pattern was recognizable even with clean bedding as stimulus. Thus, the first aim of our LFP analysis was to identify the neuronal activity under two conditions (Fig. 2a): non-exploration (left, pre-stimulus; right, post-stimulus) and exploration behaviours (middle). As exemplified in the Fig. 2a, the oscillations in non-exploration condition revealed predominant activity in delta waves (<2 Hz), with short incursions in higher frequencies (Fig. 2a-left, b). However, the exploratory behaviour shifted the activity pattern to theta frequencies (4–10 Hz; Fig. 2a–middle-, c). When the stimulus was removed and the exploratory movements decreased, the predominant frequencies fell gradually to slow pre-stimulus values (Fig. 2a–right-). Furthermore, we detected, within the exploration periods, short rhythmic epochs (2–4 s) where the oscillation temporally remained in a stationary theta band (Fig. 2a,c -in red-) apparently accompanied by an increase of the gamma activity (Fig. 2a -white brackets-). We identified these short rhythmic epochs as episodes of *sniffing-like* behaviour. This isolated theta pattern in the AOB recordings matched with the exploration of the stimulus area, even when a neutral stimulus (clean bedding) was presented (Fig. 2a,c).

**Figure 3:**
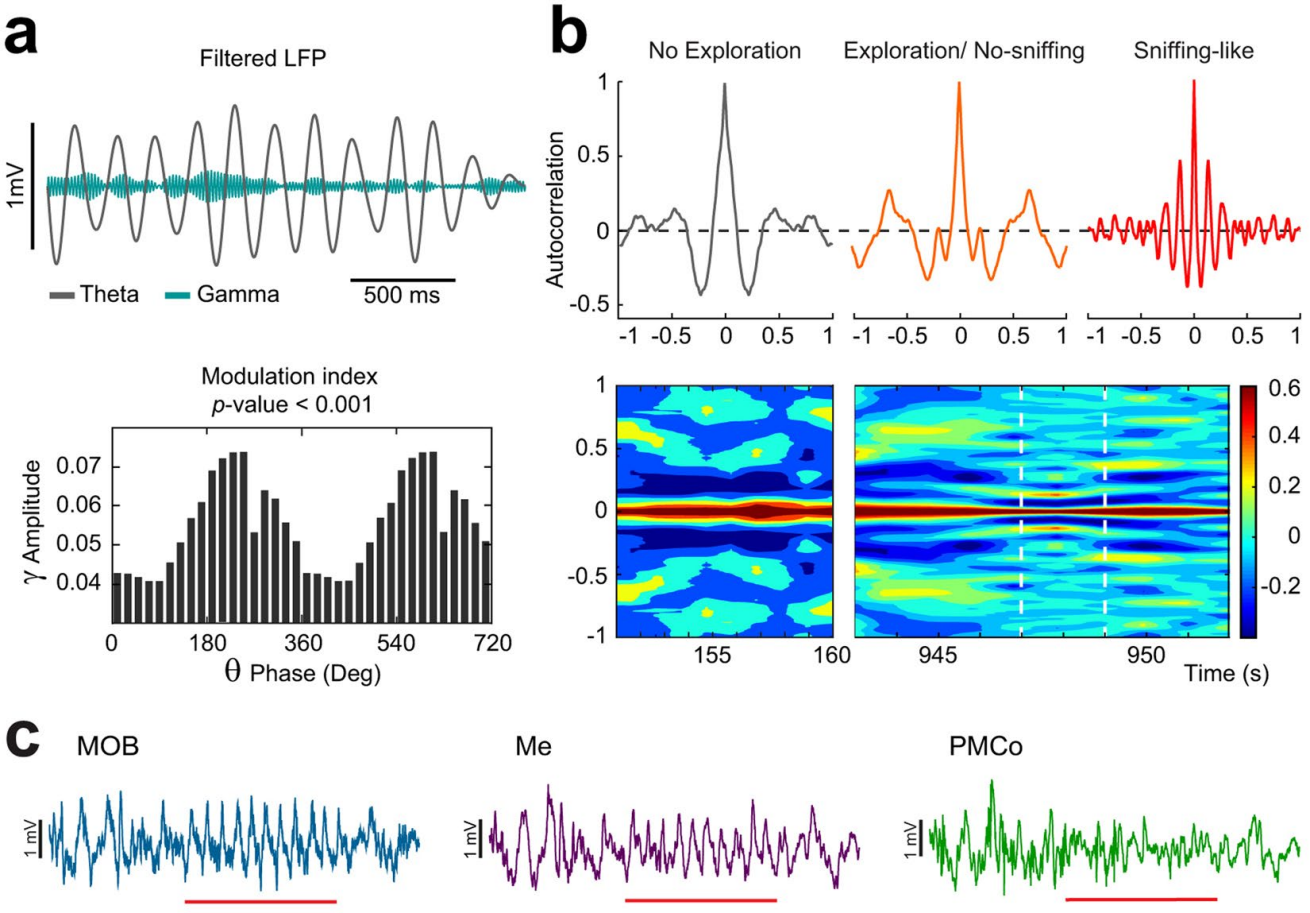
Example of a sniffing-like time in the AOB. (**a)** Up: filtered LFP for theta (3–10 Hz, black) and gamma (58–68 Hz, green) frequency bands; Bottom: phase-amplitude coupling with the oscillatory component of 58 and 68 Hz as a function of the waveform-based theta cycle phases (bin, 20°; peak of theta cycle, 90°). (**b)** Up left: autocorrelation of a non-exploration period; Up middle: autocorrelation of a period of exploration without sniffing; Up right: autocorrelation of a sniffing-like period; Bottom: evolution of the autocorrelation through time, the dashed white lines mark the sniffing*-*like period. Colour bar indicates the autocorrelation value. (**c)** Example of LFP traces recorded during an exploration time with the sniffing-like time marked with red line in the MOB, the Me and the PMCo.

We then wanted to distinguish the specific features of this pattern within the exploratory periods in the AOB. First, we represented the distribution of the amplitudes from the raw signal. A measure of the symmetry of this distribution evidenced differences between non-exploration and exploration periods. In non-exploration epochs, the amplitude distribution showed a slight asymmetry centred at positive values (skewness = −0.13 ± 0.43; Fig. 2d). In contrast, during exploration periods, the distribution showed a bimodal shape with a major peak for negative amplitudes and a minor peak in the positive counterpart (skewness = − 0.101 ± 0.24; Fig. 2e). This bimodal shape was more evident during the sniffing-like theta epochs (skewness = 0.34 ± 0.09; Fig. 2f). The asymmetry in the amplitude distribution indicates different temporal length of the negative and positive phases of the theta cycle. This suggests that, under olfactory exploration, the theta cycles contain faster waves incrusted in its negative phase, as a putative sign of coupling between different rhythmical components. An overlapped representation of the filtered signals in the theta and gamma bands suggested the phase coupling of both components (Fig. 3a, top). The coupling between the amplitude of gamma oscillations with the phases defined in the theta band could be determined by means of the modulation index (MI). The MI showed significant values in the descendent part of the theta phase (Fig. 3a, bottom).

The rhythmic nature of this sniffing-like oscillation was also characterized by periodicity measures. The autocorrelation of the raw signal in the sniffing-like periods showed high values in comparison with the non exploration as well as with non-sniffing-like exploration epochs (Fig. 3b-top-). The time-course of the autocorrelation clearly demarks the entrance in a sniffing-like epoch with high autocorrelation values periodically distributed (Fig. 3b-bottom). These measures, both the asymmetrical distribution and the rhythmicity profile, helped us to identify the short theta segments correlated with chemoexploratory behaviour.

Thus, theta bouts, identified here as short epochs in AOB during exploratory behaviours of the stimulus area, are compatible with previous descriptions of the LFP recordings in the MOB, where the olfactory activity is characterized by a sniffing process coupled to the respiratory rhythm^25^. The other recording nodes in our experiments (MOB, Me and PMCo) also showed a similar profile of activity in the exploration periods, in which the olfactory pattern previously described could be distinguished (Fig. 3c).

**Figure 4:**
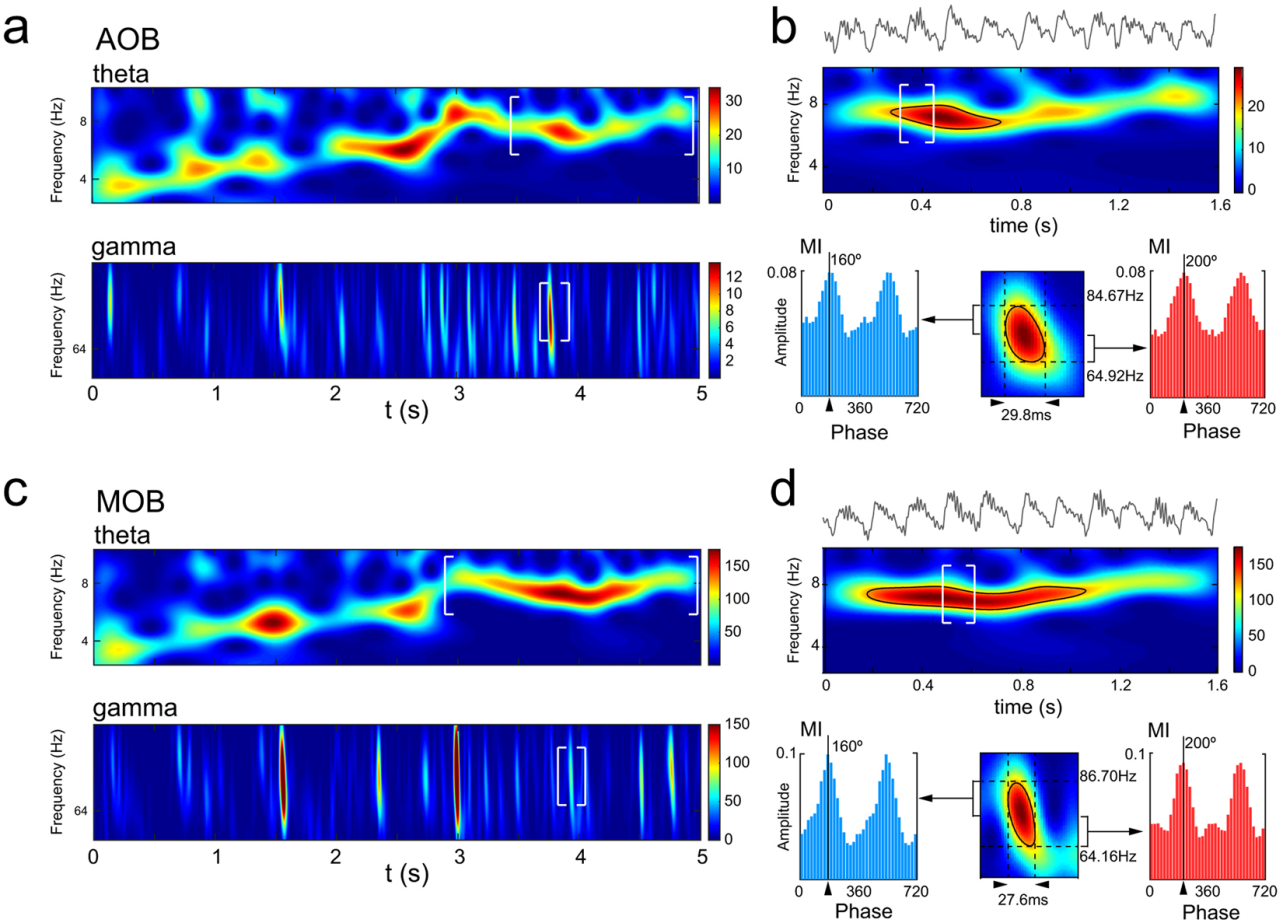
Olfactory-induced theta is associated with high and low gamma-oscillations in the AOB and MOB. (**a)** spectrogram of the theta (top) and gamma (bottom) bands; (**b**) Top, LFP trace and wavelet spectrogram of a sniffing-like period in the AOB (delimited with white brackets in (**a**). Colour bar indicates the wavelet power coefficients. Bottom: high to low gamma-oscillations wavelet analysis during theta-oscillation, delimited in white brackets in (**a**) (bottom) and (**b**) (top). High gamma is amplitude-phase coupled with the theta cycle phases at a prior phase than the low gamma. (**c)** and **(d)** Idem in the MOB.

In recent papers, a particular shift of gamma frequencies with time in MOB has been reported^26^. Here we have recognised the same patterns of the time course of gamma waves (range, 80–60 Hz), coupled to theta waves, in our MOB recordings (Fig. 4b). Moreover, this same pattern was also detected in the AOB signal (Fig. 4a), where a sniffing event was able to induce a similar gamma shift (approx. 80–60 Hz), in a different time window of the theta period, with high-gamma waves followed by a short low-gamma epoch (Fig. 4). Both gamma bands showed significant coupling with theta oscillations (MI, phase-amplitude coupling). This profile is visible in the raw signal of both areas, where the descendent phase of theta cycle is overlapped with fast oscillations.

### Analysis of the theta and gamma oscillatory pattern in the recorded structures

**Figure 5:**
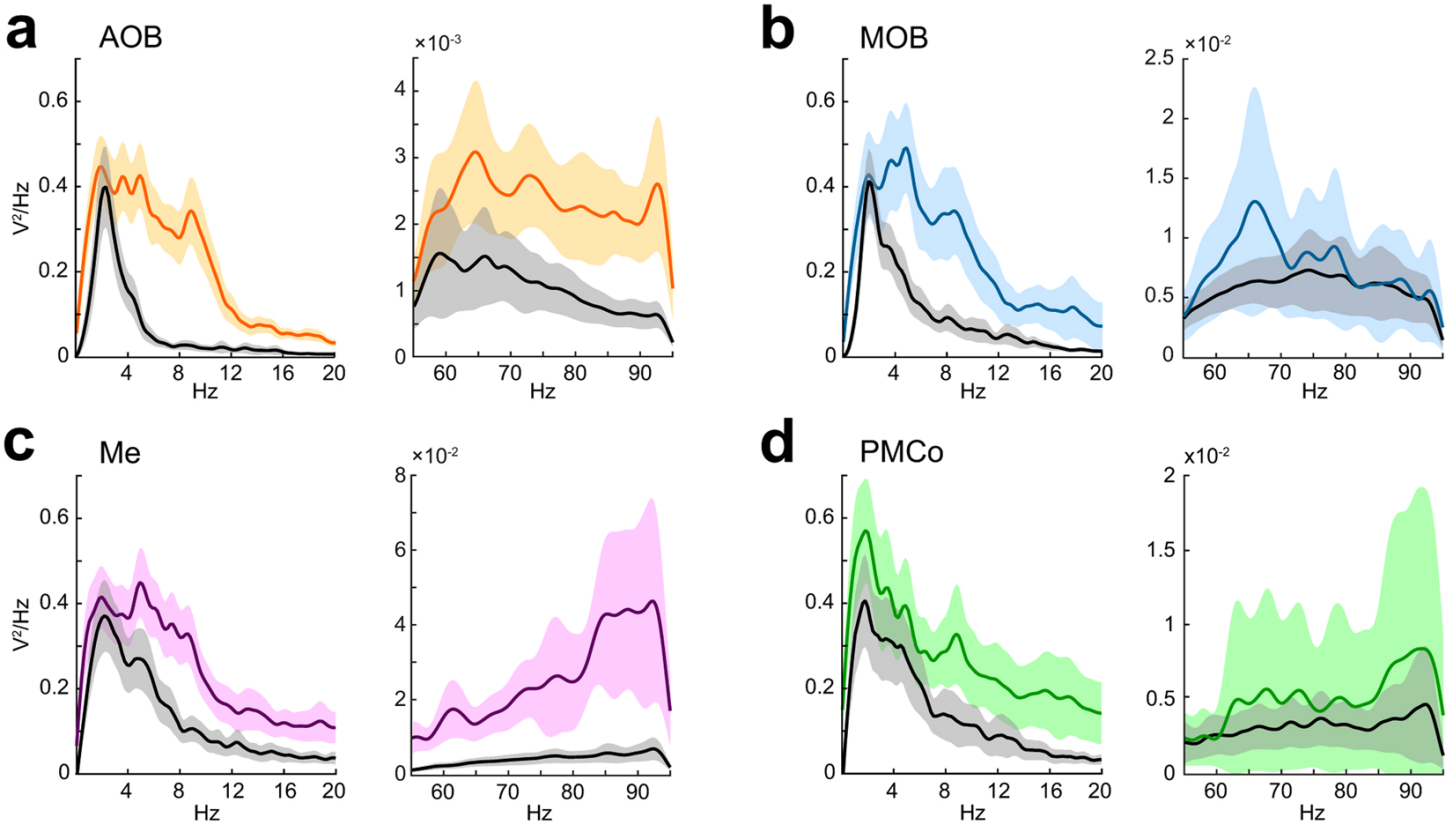
Olfactory-induced changes in the recorded nuclei. (**a**) Power spectra in the AOB for clean bedding (in colour) and non-exploration (in black), showing the average (solid line) and standard deviation (shadowed area) for the 0–20 Hz frequency band (left) and 55–95 Hz frequency band (right). In the MOB (**b**); the Me (**c**) and the PMCo (**d**).

We analysed the neural response of the olfactory circuit with the exploration of a neutral stimulus (clean bedding). The power spectral analysis displayed a prominent theta activity with a peak at 4–6 Hz in the four target areas, according to the above description (Fig. 2). However, a specific analysis of the gamma waves evidenced clear differences between the recording nodes in the sniffing-like periods. Whereas AOB and MOB showed an increase of gamma power (60–80 Hz; Fig. 5a,b), the AOB, Me and PMCo displayed prominent power in the higher gamma frequencies (85–95 Hz; Fig. 5a,c,d). A particular analysis of the power spectra showing the effect of conspecific stimuli is displayed in Supplementary Fig. 2. Under these conditions, the peak of theta waves in all four recorded areas shifted to higher values with regard to neutral cues. Furthermore, whereas the gamma band showed a similar spectral distribution in all the nuclei, the AOB power spectrum in the gamma band showed a clear power increase (Supplementary Fig. 2a).

In a further step, we next carried out a detailed analysis of the spectral distributions comparing exploration and non-exploration epochs (see Supplementary Fig. 3 and Supplementary Table 1). The data of the different stimuli were averaged weighted for the number of exploration periods that each animal performed. Overall, when analysing the wide frequency range (0.5–95 Hz) we observed a distinctive demeanour in the Me as compared to the other nuclei (Supplementary Fig. 3a). Generally, the exploration induced a significant increase in the power ratio of the theta band accompanied by a decrease in the delta waves. Both bulbs did not exhibit significant differences in their gamma distributions, although the comparisons of the mean value of the 30–40 Hz band had a *p*-value = 0.06 in the MOB (Supplementary Table 1). The PMCo activity followed a theta-band increase correlated to that of AOB but no delta decrease (Supplementary Table 1). Remarkably, Me showed an increase in the power ratio of the gamma frequency segments during the exploration behaviours, while the delta and theta bands decreased their powers (Supplementary Fig. 3a–Me-); thus indicating that the Me presents theta waves accompanied with a more relevant gamma in comparison to the other recorded nuclei. The analysis of the theta (relative to 0.5–30 Hz power ratio) and gamma (relative to 30–95 Hz power ratio) allowed us a better understanding of the oscillations taking place during olfactory processing (Supplementary Fig. 3b). According to the power spectra (Fig. 5), two distinctive gamma bands showed a different behaviour. Whereas the AOB evidenced an increase in the gamma waves (>90 Hz) during the exploration, the MOB showed differences in a lower gamma range (60–80 Hz) (Supplementary Table 1). Meanwhile the amygdaloid nuclei showed an increase in activity in the gamma band, although in the PMCo it does not reach statistical significance (p = 0.08) (Supplementary Table 1). Altogether, these results suggest a differential gamma profile between nodes correlated to the olfactory theta activity.

Overall, local gamma frequency simultaneously increased its power, in each of the recorded areas, as a response to the stimulus exploration. We observed that the entrance in a sniffing-like epoch, inside an exploration period, is accompanied by a shift of the corresponding theta peak and an increase of the gamma power (Supplementary Fig. 4). Previous to the sniffing-like event, the gamma peak remained below significant levels (95% confidence level), in contrast to the higher peaks above the significant line under the olfactory activation.

### Differential theta rhythmicity under the exploration of neutral and conspecific stimuli

**Figure 6:**
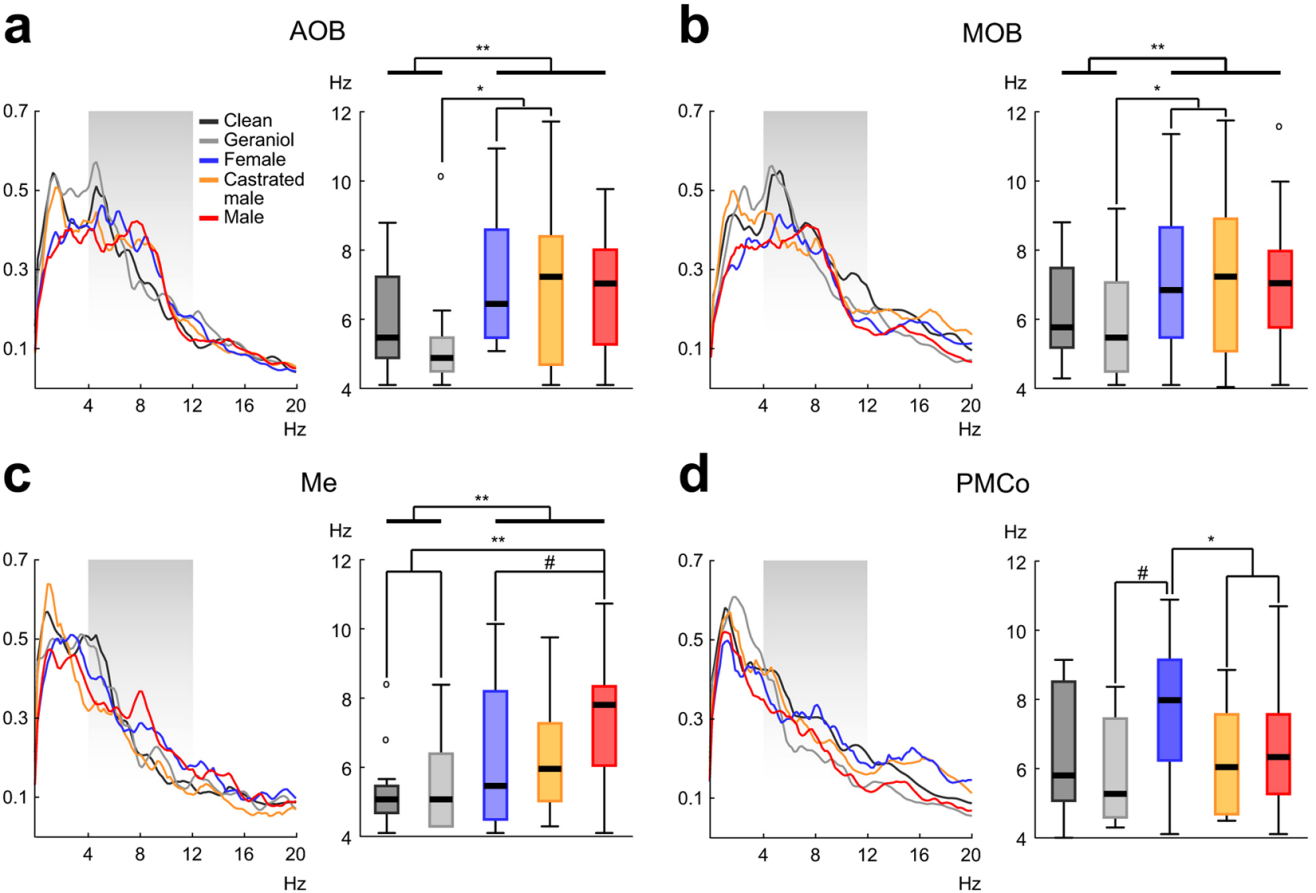
Different stimuli induce unalike/distinctive peaks in the theta range. (**a)** Left: mean normalized power spectra of the AOB field recordings, the shaded area points the *theta* frequency band; Right: main peak distribution for the different stimuli, the thick lines show the comparison between the neutral and the conspecific derived stimuli (^#^p-value ∼ 0.05; *p-value < 0.05; **p-value < 0.01). Idem for the MOB (**b**), the Me (**c**) and the PMCo (**d**).

The spectral profile of the neural activity showed a distinctive theta pattern with the exploration of specific stimuli (left panels in Fig. 6). As we described above, olfactory exploration leads to a prominent theta activity in the recorded sites, indicative of a sniffing-like process (right panels in Fig. 6). With these data, we performed comparisons of the theta dominance for each recorded node and between stimuli: pair-wise comparisons of all the stimuli; neutral vs. the conspecific-derived stimuli; and among the conspecific stimuli.

In the AOB, (Fig. 6a), the main theta peak was different among stimuli (*K* = 12.042; *p* = 0.017). Pairwise comparisons revealed significant differences between the geraniol-scented bedding and the castrated (*p* = 0.044) and female-soiled bedding (*p* = 0.041); and differences, but non-significant, between the geraniol-scented bedding and the male-soiled bedding (*p* = 0.058). A similar result was obtained in the MOB (Fig. 6b), where the comparison showed differences among stimuli (*K* = 11.822; *p* = 0.019) with pairwise differences between the geraniol-scented bedding and the castrated (*p* = 0.044) and female-soiled bedding (*p* = 0.038). The comparisons in the Me also showed clear differences (Fig. 6c; *K* = 18.205; *p* = 0.001) where male-soiled bedding was different from the clean (*p* = 0.007) and from geraniol-scented bedding (*p* = 0.01). In the case of the PMCo (Fig. 6d), the statistical analysis revealed significant differences in the broad comparison (*K* = 11.578; *p* = 0.021), but no differences in the pairwise comparisons.

Comparing the neutral with the conspecific-derived stimuli, significant differences in the AOB were demonstrated (*U* = 903.0, *p* = 0.001), where the neutral stimuli had a mean theta peak of 5.74 ± 0.31 Hz, while for the conspecific-derived stimuli the mean peak remained around 6.88 ± 0.18 Hz. The MOB also showed such differences (*U* = 2453.0, *p* = 0.001), where the neutral and conspecific-derived stimuli had mean peaks with similar values to that of AOB (6.04 ± 0.15 Hz and 6.99 ± 0.20 Hz, respectively). In the Me recordings, the mean frequency reached 5.4 ± 0.23 Hz with the neutral stimuli and 6.85 ± 0.22 Hz for the conspecific stimuli (U = 592.0, *p* = 0.001). No statistical differences were observed in the PMCo (Fig. 6d), where the peaks were 6.19 ± 0.33 Hz (neutral) and 6.81 ± 0.19 Hz (conspecific-derived stimuli).

Finally, the comparison among the conspecific-derived stimuli revealed no differences in the olfactory bulbs, but significant differences appeared in the amygdaloid nuclei. The Me showed differences in the conspecific comparison (*K* = 6.581; *p* = 0.037), but no differences in the pairwise comparisons (Fig. 6c), although the female to male-soiled bedding was close to significance (6.42 Hz and 7.39 Hz, respectively; *p* = 0.065). Furthermore, the analysis for the PMCo showed differences in the general comparison (*K* = 8.793; *p* = 0.012) and in the pairwise comparisons (Fig. 6d), with significant differences between the female and the castrated (7.69 Hz and 6.27 Hz, respectively; *p* = 0.02) and male-soiled beddings (6.54 Hz; *p* = 0.042). Overall, these data suggest that the olfactory exploration leads to a particular theta oscillation whose profile is different attending to the node of the olfactory network and evidenced by specific stimuli.

As described before, the PMCo power spectra showed a non-significant peak (*p* = 0.08) in the high-gamma band when exploring clean-bedding. Since the PMCo responds in a particular manner to female cues, we performed an analysis of the power ratio comparing the female and the non-exploration within the gamma range (30–95 Hz). This result reveals now a significant increase in the high-gamma oscillations (*W* = 2.023; *p* = 0.043).

### Synchrony between neuronal populations of the olfactory network

The presence of theta oscillations in the olfactory network under exploration suggests that this activity could be the neural correlate of communication among structures. For this purpose, we measured the phase synchronization between pairs of signals by means of the phase-locking value (PLV, 0–1 range) and phase lag index (PLI, 0–1 range). The phase analysis was performed filtering the raw signals at the main theta peak frequency (1 Hz wide) as calculated during the comparison of the stimuli.

Regarding the synchronization between the olfactory bulbs, the time course of the PLV for a representative case with exploration and non-exploration periods is showed in Supplementary Fig. 5a. At the first stretch of the time course plot, low values of synchrony could be observed in a non-exploration period (Supplementary Fig. 5c), attending to the behaviour of the animal. In contrast, during exploration, the phase locking increased (Supplementary Fig. 5b). Within this exploration period, the synchronization showed high values of both PLV and PLI in segments corresponding to a sniffing-like pattern (Supplementary Fig. 5b,d). A study of the synchronization values in the AOB and MOB showed significant differences between periods. Whereas during non-exploration periods the PLVs values in the theta band remained in low levels (0.55 ± 0.19), the investigation of geraniol-scented bedding increased the synchronization (PLV, 0.93 ± 0.07; PLI, 0.94 ± 0.08) during the sniffing-like periods (*U* = 13.00; *p* < 0.01). However, in both periods, the Rayleigh’s test demonstrated a phase preference between signals (control: *z* = 426.75, *p* < 0.05; sniffing: *z* = 767.49, *p* < 0.05), suggesting that, even during non-exploration periods, there is a temporal relationship between the two signals.

The synchronization between the AOB and the Me showed relatively low values (0.50 ± 0.23) in the non-exploration times which significantly increased during the sniffing-like epochs (PLV, 0.96 ± 0.03; PLI 0.97 ± 0.06; *U* = 1.50; *p* < 0.01). As described before, in this case the Rayleigh’s test was significant in both during non-exploration (*z* = 577.90; *p* < 0.05) and sniffing periods (*z* = 1393.07; p < 0.05).

In the case of the coupling between the AOB and the PMCo, low PLVs were observed in the control periods (0.43 ± 0.13) and they significantly increased in the sniffing-like periods (PLV, 0.89 ± 0.09; PLI 0.94 ± 0.09; *U* = 1.00; *p* < 0.01). According to the Rayleigh’s test (non-exploration: *z* = 451.76, *p* < 0.05; sniffing: *z* = 680.41, *p* < 0.05) phase-preference was present in both conditions.

**Figure 7:**
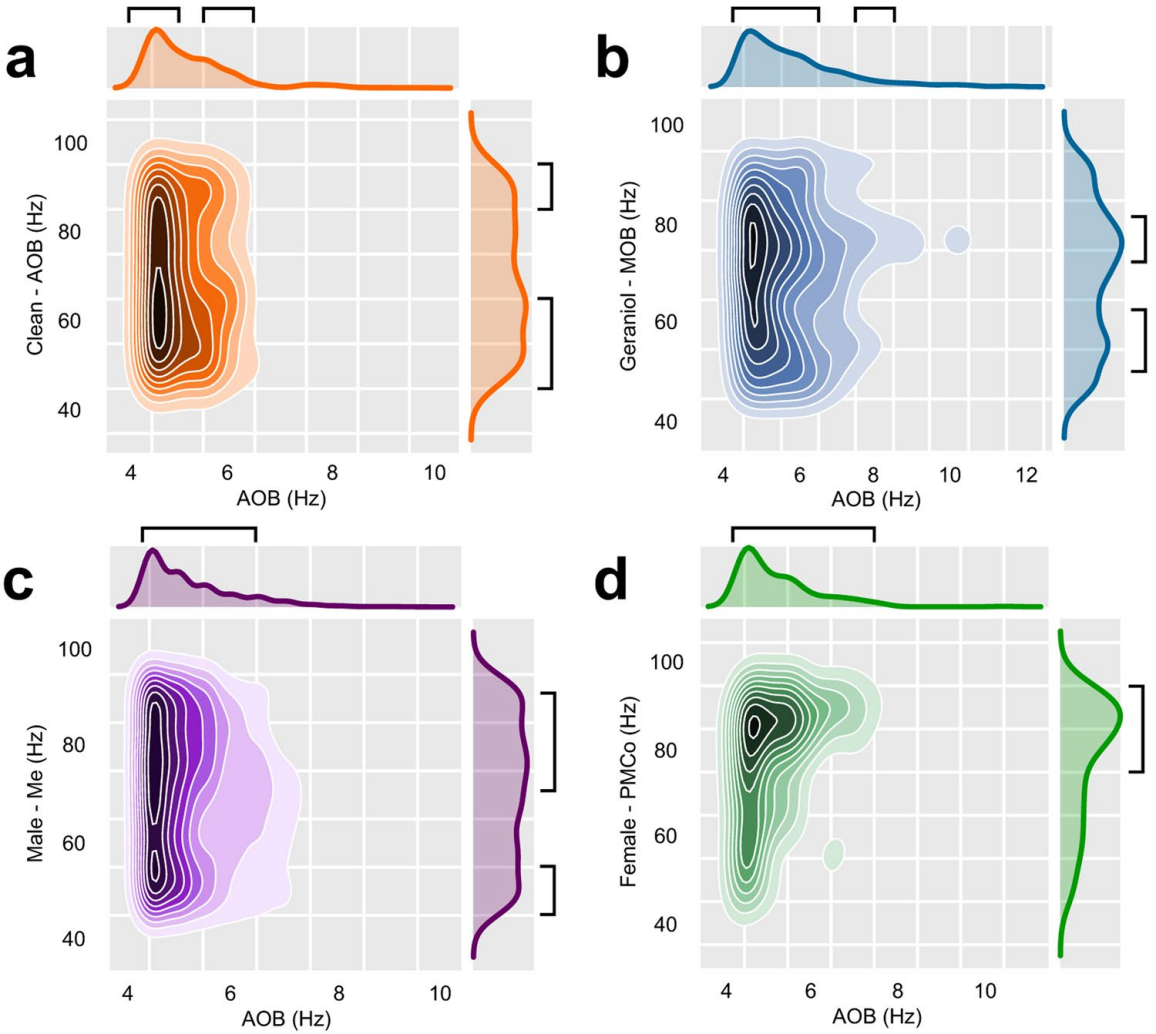
Synchrony between the AOB and the AOB itself, the MOB, Me and PMCo. (**a**) Density plots of the points of modulation in the gamma band (AOB) induced by the theta frequencies (AOB) for clean bedding, calculated as the mean of the modulation values for all times in each recording, only values over the 70^th^ percentile are shown. (**b**) Idem for the MOB, geraniol-scented bedding. (**c**) Idem for the Me, male-soiled bedding, showing the over 65^th^ percentile values. (**d**) Idem for the PMCo, female-soiled bedding, showing the over 70^th^ percentile values. Black brackets indicate the regions of interest.

As we described above, gamma bursts could be modulated by theta waves. To examine whether this modulation is present between recording nodes, we assessed the phase-amplitude coupling between different frequencies and/or signals. For our study, we detected the phase of theta cycles in the AOB (1 Hz range) modulating the amplitude of the gamma (10 Hz range) oscillation in the AOB itself and in the other nuclei. The frequencies around 4–5 Hz showed a very high modulation over a wide range of gamma-frequencies (55–95), with a peak of modulation around 60 Hz, and a second zone of modulation around 90 Hz (Fig. 7a).

Concerning the interrelation between AOB and MOB (Fig. 7b), theta frequencies up to 6 Hz in the AOB overlapped with gamma oscillations (50–85 Hz); an additional zone of modulation was detected in 70–80 Hz band coupled to 8 Hz theta oscillations. In the Me the range of AOB’s theta frequencies modulating gamma oscillations was larger (Fig. 7c). The modulation by frequencies between 4–6 Hz is associated with two gamma regions at 55 and 70–85 Hz. In the PMCo, the range of theta-gamma modulation was similar to that of the Me (Fig. 7d), but the gamma regions were clearly higher (>85 Hz).

These results are consistent with the previously described power ratio analysis, suggesting a differential gamma profile between nodes correlated to their theta activity.

## Discussion

The present work investigates in awake, freely behaving female mice, the activity of the vomeronasal system induced by the exposure to neutral odorants (clean bedding and geraniol-scented bedding), or conspecific chemosignals (male- and female-soiled bedding, and bedding soiled by castrated males). For comparison, the experiments include simultaneous analysis of the activity in the MOB. As reported previously^27^, the stimuli derived from conspecifics elicited a higher level of chemosensory investigation.

Surprisingly, the results reveal that during exploratory behaviour oscillatory activity in the vomeronasal system shows a clear theta rhythm even when only neutral olfactory stimuli are present, without vomeronasal cues. In contrast, in the absence of exploratory behaviour, field activity in the delta frequencies (<4 Hz) predominates. The power of the theta oscillations observed during exploratory behaviour increases when the animals apparently focus on chemoinvestigation, and we identified, within these periods, episodes of sniffing-like behaviour. In the AOB, during these sniffing-like periods, the theta rhythm modulates particular gamma oscillations, located in the peak and descending parts of the theta cycle. Our results show that this gamma oscillation has a particular shift from ∼80 Hz to ∼60 Hz, where high gamma component appears in the descending phase of the theta cycle, followed by the low gamma component. This is very similar to description of gamma component described in the MOB associated to the odour response^26, 28^. We have compared this gamma shift in the MOB and AOB, and show that they have similar frequency components but are located in different time windows. This gamma shift has been shown in the MOB to be associated with the inhalation-exhalation transition of the respiratory cycle^26, 28^. Our results, including the phase difference of the theta rhythmicity of the AOB and MOB, suggests that the gamma shift in the AOB, although coupled to the MOB activity, take place in a different time of the respiratory cycle.

The theta oscillatory activity observed in the AOB was similar in the MOB, suggesting that the activity of both centres is synchronic during exploratory behaviour of olfactory stimuli. A recent description about the functional link between both olfactory bulbs shows physiological proves of the presence of axonal collaterals from the principal cells of the AOB to MOB neurons^29^.

**Figure 8:**
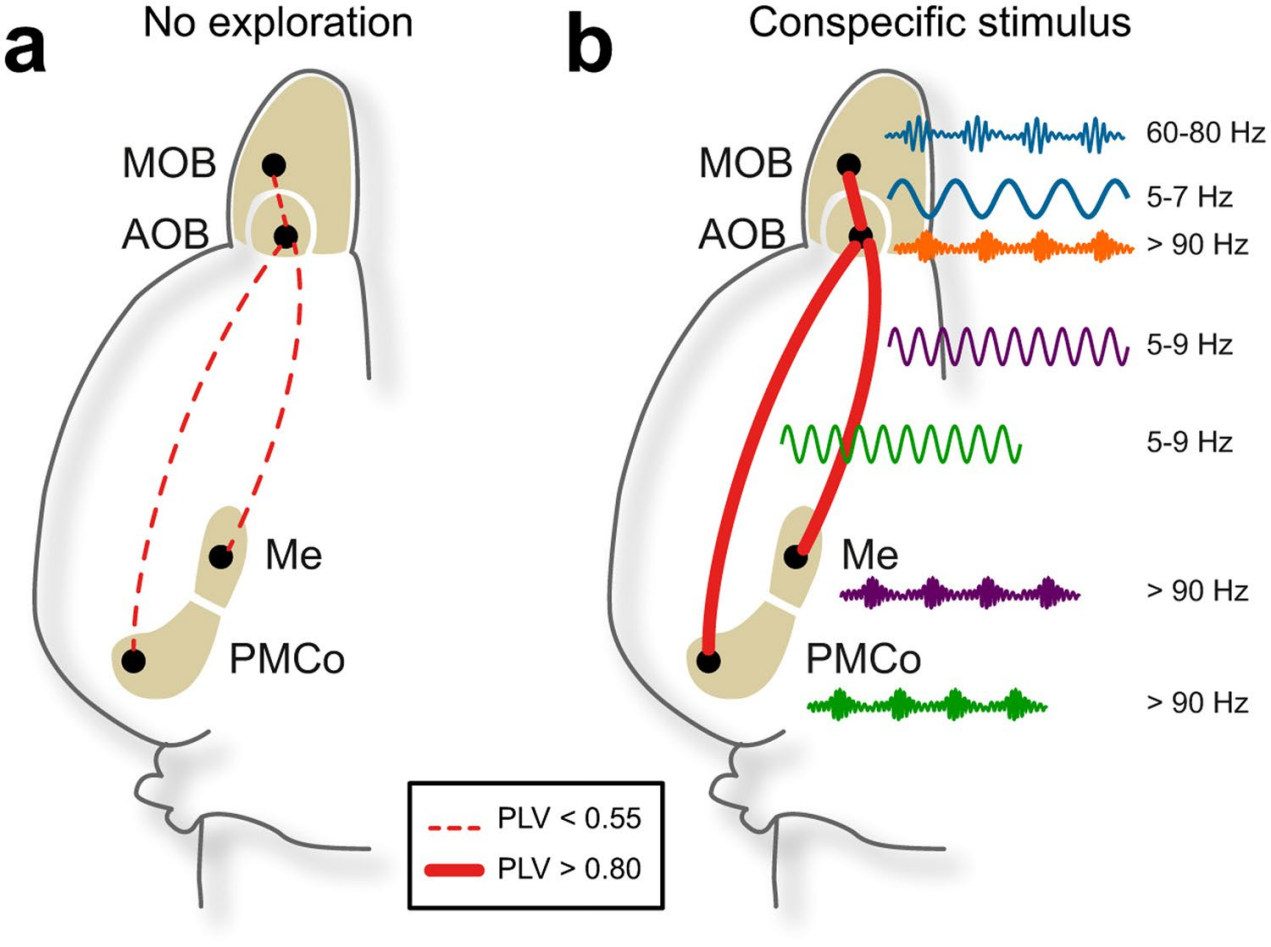
Long-range interactions in the olfactory network. (**a)** Default network, synchrony level and representative LFP filtered in the dominant frequencies in non-exploration conditions. (**b**) Synchrony levels for neutral stimulus (AOB/ MOB) and conspecific stimuli (AOB/ Me and AOB/ PMCo) and representative LFP in the dominant theta and dominant gamma frequencies in exploration conditions.

There are several reasons to discard the possibility of volumetric conduction of the LFP signals from the MOB to the AOB. On the one hand, apart from the described peak in the theta band, the distribution of other frequency peaks in the power spectra is different. In addition, the phase-locking value is very low when the animals are not engaged in chemoexploratory behaviour (Fig. 8a). Moreover, we have included in our results the *phase lag index* as a robust parameter of phase-locking measure that discards 0 *mod* π phases, and so excluding volumetric conduction noise. In periods of sniffing-like oscillations the values of both PLV and PLI are close to 1, indicating very high coupling with independent recordings.

During the exploration of geraniol-scented bedding by means of sniffing-like behaviour, the MOB and AOB show a very high degree of coupling in the theta band (Fig. 8b), and the theta activity in the AOB is related to gamma oscillations in the MOB. These results further reinforce the idea that the activity of the MOB and AOB is synchronized even during the detection of pure olfactory stimuli, as shown previously using high resolution fMRI in mice^30^. Finally, synchronic activity of the main and accessory olfactory systems has also been reported in rats^22^. The coupled oscillatory waves between the different nodes of the olfactory network, probably reflects a temporal coordination of the detected chemosensory information.

In the gamma frequency bands, the analysis revealed differences between the MOB and the vomeronasal nuclei. In the AOB, Me and PMCo, the stimulus exploration gives rise to activity in the high-gamma band, different from the low-gamma profile in the MOB. When the activity in the gamma frequency band is compared in the periods of exploratory activity, induced by the different stimuli (versus periods of no exploration), the medial amygdala stands out as the structure showing higher levels in these frequency bands. The activity in the gamma bands is thought to reflect local processing^31^, and in this regard, the local microcircuitry of the medial amygdala is very different from that of the bulbs and the PMCo. Whereas the olfactory bulbs and the PMCo are structures of major pallial origin^32^, with glutamatergic projection neurons and inhibitory interneurons, the medial amygdala is a subpallial structure containing neurons of different embryological origin, including neurons originated in the caudal medial ganglionic eminence, in the preoptic region and in the hypothalamic supraoptoparaventricular domain, as well as neurons from the adjacent ventral pallium^33–35^. Part of the projection neurons of the medial amygdala are GABAergic^36^. Therefore, the local processing in the intrinsic circuits of the medial amygdala does not follow the same organization observed in pallial structures. The differences in gamma activity in the medial amygdala when compared to the bulbs or the PMCo may be related to the existence of different populations of interneurons. These results are consistent with the PMCo being the primary vomeronasal cortex^37^, while the medial amygdala corresponds to the striato-pallidal output station^38, 39^.

In the main olfactory system, the rhythm of odour-sampling behaviour (sniffing) strongly drives the activity in the MOB^25^. In addition, sniffing pattern is also influenced by social stimuli^40^. The neural control of sniffing depends on snout muscles innervated by motor neurons of the facial nerve^41^, which in turn receive projections from the pre-Botzinger, Bötzinger complex and parafacial respiratory group^42^. This complex includes the central pattern generator of the respiratory rhythm.

On the other hand, vomeronasal pumping is under autonomic control via sympathetic neurons of the superior cervical ganglion^23^, whose preganglionic neurons are in the intermediolateral column of the cervical-thoracic levels of the spinal cord^43^. These spinal preganglionic neurons receive important projections from the lateral hypothalamus^44^, which in turn is innervated by the Pir, the anterior olfactory nucleus, the olfactory tubercle and the anterior cortical amygdala^45^. Therefore, the detection of olfactory information may activate the vomeronasal pumping by this direct hypothalamo-spinal projection. A similar circuit through the lateral hypothalamus has been anatomically described in snakes^46^, in this case to mediate the chemosensory modulation of the tongue-flick response, the mechanism for internalization of chemosignals into the vomeronasal organ^47^. The detection of airborne odours increases tongue-flick rate in snakes^48^, suggesting that the role of the main olfactory system in activating vomeronasal sampling is general in terrestrial vertebrates. The lateral hypothalamus projects also to the rostral ventrolateral reticular nucleus^44^, where the pre-Botzinger and Bötzinger complex are located. Consequently, the lateral hypothalamus may coordinate the detection of olfactory information with both olfactory (sniffing) and vomeronasal (vascular pumping) internalization mechanisms^49^, explaining the coupled oscillatory activity. In this regard, vomeronasal pumping in male hamsters is activated by novelty, and keeps active during exploratory behaviour^50^. Furthermore, lesions of the olfactory epithelium with zinc sulphate, leaving intact the vomeronasal organ, disturb vomeronasal-dependent behaviours^51^, such as intermale aggression^52^. These results also indicate that olfactory stimuli trigger the activation of the vomeronasal pumping^51^. Therefore, the profound behavioural deficits observed in some mice with null mutations of genes coding for essential proteins for olfactory signal transduction (e.g., Mandiyan *et al*.^53^) may be reinterpreted as the result of the inability to detect both olfactory and vomeronasal information. Previous works have shown that combined olfactory and vomeronasal lesions or combined genetic alterations induced more severe behavioural deficits that either selective olfactory or selective vomeronasal disruptions^54^.

The conspecific-derived stimuli induced particular changes in the oscillatory activity of the analysed circuits. In the theta band, the investigation of chemical signals from either females, males or castrated males induced oscillatory activity at approximately 7 Hz in all four structures analysed. In contrast, as discussed before, the exploration of neutral stimuli induced theta activity at 4–6 Hz. The differences in the predominant theta induced by olfactory stimuli versus those induced by conspecific stimuli are significant in the AOB, MOB and Me, and approach significance in the PMCo. These results are similar to those reported by Tendler and Wagner^22^ in the olfactory bulbs and medial amygdala (they did not record in the PMCo) in a social recognition paradigm, although they uses juvenile male rats as subjects, and either awake or anesthetized male rats of similar age and different strain as stimuli. The present results show that chemosensory cues from conspecifics are enough to induce this oscillatory activity, which appears common to rodents and independent of the gender of the subjects. In the main olfactory system it has been suggested that lower frequency activities might be attributable (in addition to the respiratory cycle) to inhibitory activities from the anterior piriform cortex or anterior olfactory nucleus^55^. Similarly, in the case of the AOB, it may be mediated by feedback signals from the PMCo^37^ or medial amygdala^38^.

In the MOB and AOB, the main theta peak induced by female- and castrated male-soiled bedding is also significantly different from that induced by geraniol. The theta rhythm induced by male-soiled bedding is similar to that elicited by female and castrated male cues. The lack of differences in the AOB activity induced by male- or female-derived chemical signals is consistent with the results of multiunit and single unit recordings^56, 57^, which show a relatively low selectivity of AOB responses, since some neurons respond to different types of stimuli. The presence of vomeronasal sensory neurons with broadly tuned responses, able to detect all of the major urinary proteins tested^58^, may be the reason underlying this low selectivity.

In contrast to the AOB, in the medial amygdala male-specific signals elicited a rhythmic theta activity with a peak near 8 Hz, different from that induced by female chemicals. Among the chemical signals that may elicit this distinct activity, two non-volatile pheromones are strong candidates. On the one hand, the male-specific major urinary protein darcin, which elicits female attraction^4^, olfactory^59^ and spatial learning^60^, and maternal aggression^61^; and, on the other hand, the peptide ESP-1, secreted by the extraorbital gland of male mice, which induces lordosis^3, 62^. Both darcin and ESP-1 are detected by vomeronasal sensory neurons (darcin^58^; ESP-1^3^). The present results suggest that the medial amygdala is a key nucleus in processing male-derived sexual signals, consistent with previous electrophysiological^57^, c-Fos^63^ and lesion studies^64^.

In addition to darcin and ESP-1, several male-specific volatile molecules influence sexual behaviour in females^65^, such as the vomeronasally-detected farnesenes, brevicomin and tiazoline^66^ and (methylthio)methanethiol (MTMT) and (Z)-5-tetradecen-1-ol, detected by the olfactory epithelium^67, 68^. The vomeronasal signals may obviously contribute to the male-specific activity of the Me. In addition, olfactory information also reaches the Me directly from the MOB^10^ and indirectly from the piriform cortex and the olfactory amygdala^39^.

The analysis of the coupling values shows also that the theta activity in the AOB and Me is strongly synchronic during the sniffing of male-soiled bedding (Fig. 8b). In addition, the theta oscillations in the AOB modulate the activity in the whole gamma band in the Me. These results are consistent with the Me processing information about male-specific vomeronasal signals.

In contrast to the Me, in the PMCo a peak of oscillatory activity in the theta band is specifically elicited by female chemosignals. The female major urinary proteins also play a role in female aggressive behaviour, in the context of access to resources or reproductive opportunities^69^. In addition, a recent female-specific urinary signal named cortigynic acid has been identified^70^. This molecule is a non-volatile vomeronasal signal that elicits chemoinvestigation and induces mounting behaviour in males^70^. Although it is not currently known whether cortigynic acid is detected by females, it would be interesting to investigate the role of the PMCo in processing female-derived vomeronasal signals.

The analysis of theta coupling among the AOB and PMCo during the exploration of female stimuli revealed a strongly synchronic activity in these two structures (Fig. 8b). Moreover, our data also indicate that theta activity in the AOB is related to the high gamma oscillations in the PMCo.

Altogether, the synchronic theta activity between the MOB and AOB, and between the AOB and the vomeronasal amygdala, suggests that this theta rhythm, as showed for other brain regions, facilitates the coordination of the network activity for the transfer of information across these brain regions. Hierarchical operations in the olfactory network could explain the presence of a common theta oscillator with a feedforward processing between the olfactory bulbs and the amygdaloid nodes, and in this way, particular gamma oscillations emerge in the local circuits with fine time-scale coordination. Thus, olfactory and vomeronasal information could be integrated into a complete representation of the chemosensory environment.

## Material and Methods

### Subjects

The animals used in the experiment were 9 adults, virgin female mice (*Mus musculus*) of the CD-1 strain (Janvier, France). Estrous cycle was not controlled. Adult mice of the same strain provided urine-soiled bedding material (soft wood shavings, Souralit S.L., ref. 3000, Spain). All animals were housed at 23 °C with a natural light cycle and food and water were available *ad libitum*. The experimental procedures were approved by the Research Ethics and Animal Welfare Committee of the University of Valencia (A1431417790135 and A1283764105250) and are in accordance with European Communities Council Directive (2010/63/EU) on the protection of animals used for scientific purposes.

### Surgical and post-surgical procedures

The electrode implantation was performed under ketamine (75 mg/kg) - medetomidine (1 mg/kg) anesthesia, with local anesthesia (lidocaine, 5%) on the scalp and pressure points and atropine (0.05 mg/kg) to reduce cardio-respiratory depression. The anesthetized mice were secured to a stereotaxic frame (Narishige, Japan) and maintained at 37–38 °C with a heating pad. Following a midline sagittal incision, 7 trephine holes (4 electrode, 1 reference, 2 screw holes) were drilled by adapting the stereotaxic coordinates from Paxinos & Franklin^71^ to CD-1 mice (Fig. 1a). The local field potential was recorded with a stainless steel polyimide-coated macroelectrode (E363/3/SPC 0.125 mm, PlasticsOne, USA). The measure of the impedance in a 0.9% NaCl solution, with pulses at a frequency of 100 Hz, was of 13–16 MOhms. The electrodes were implanted in the accessory olfactory bulb (AP: − 4.75, L: −1, D: +2.4; at an angle of 45° to the vertical), main olfactory bulb (AP: −4.75, L: +1, D: +1.3), medial amygdaloid nucleus (AP: +1.3 to +1.4, L: −2.1, D: +5.2) and posteromedial cortical amygdaloid nucleus (AP: +2.7, L:−3, D: +5.6); antero-posterior coordinates (in mm) measured from Bregma and depth measured from cranial surface. The field potential recordings were referenced against an indifferent electrode placed in the epidural cerebellar area. The electrodes were connected to a six-channel electrode pedestal (PlasticsOne), which was fixed to the head using dental cement. After the surgery, the mice received injections of atipamezol (5·10^−4^ mg/g), to revert the anesthesia effects, and butorphanol (0.02 mg/g), as an analgesic. The animals were housed in a transparent plastic cylindrical cage (30 cm diameter) during the recovery and throughout the whole period of study, except for the experimental tests (see below). After the subjects had fully recovered (3 days), the mice were trained to be able to sustain the weight of the recording cable. To do so a screw was glued to the electrode pedestal tap on the mouse’s head and 3 nuts were gradually added in the following 4–5 days. During this period, the animals were habituated to the experimenter and the recording procedure.

### *In vivo* recordings

Each animal was sequentially exposed to the following odour stimuli, always in the same order: neutral stimuli (clean bedding and geraniol-scented bedding), conspecific-derived stimuli (castrated male-, female- and intact male-soiled bedding). Gonadally intact male mice were housed individually in a clean plastic cage to provide soiled bedding. Castrated males and female mice were housed in groups of 6 animals to provide soiled bedding. The bedding was collected after 4 days of use and frozen that same day to provide pheromonal stimulation. Geraniol-scented bedding was made by mixing artificial geraniol extract (1 μl/10 g; Ventós S.A., International Flavors and Fragrances, Spain) with clean bedding.

All recordings took place with the mouse moving freely in a methacrylate opaque box (42.5 × 26.5 × 18 cm). A control period of 20 min was recorded before the presentation of each stimulus (two trials per stimulus). For the stimulus presentation the mouse was transferred for 5 min to an identical box containing 15 ml of the stimulus, which was placed into a glass plate (6 cm diameter) to which the animals had full access. After the 5 min recording session, the animal was transferred back to the control box and allowed to rest for 20 min until the presentation of the next stimulus. The succession of control and stimulus-presentation periods was repeated until all five stimuli were presented.

Raw field activity was amplified and online-filtered between 0.3 and 300 Hz and the 50 Hz noise was analogically removed (p55, Grass Technologies; Ampli 4G21, CIBERTEC, Spain). Then the signal was digitized (Power 1401; Cambridge Electronic Design, UK) for offline analysis (400 Hz sampling frequency). The waveforms were continually monitored online using Spike 2 software (Cambridge Electronics Design).

### Evaluating the chemoexploratory behaviour

The behaviour of the animals was evaluated in the recorded videos. We scored the cumulative time that the mouse approached the glass plate and investigated each stimulus in each one of the five-minute sessions, and these data were analysed to calculate the time spent exploring the provided stimuli.

Under exploratory behaviour, the activity sometimes became very rhythmic and concentrated in the *theta* band (see Results). To properly characterize this oscillation, we double-checked the behaviour of the animal and the raw signal to accurately define the analyzed time periods. Thus, for the stimuli comparison we only considered the times where, in the video, the animal actively touched the stimulus with the snout while their whiskers moved (as a sign of *sniffing*) and the raw signal showed a rhythmical *theta*-centred oscillation.

### Histological verification

When data acquisition ended, animals were deeply anaesthetized with sodium pentobarbital (100 mg/kg; Dolethal Vetoquinol, Spain) and transcardially perfused with saline solution (0.9%) followed by 4% paraformaldehyde diluted in phosphate buffer (PB, 0.1 M, pH 7.6). Then, brains were removed, postfixed overnight in the same fixative and cryoprotected in 30% sucrose in PB (0.1 M, pH 7.6) at 4 °C until they sank. We used a freezing microtome to obtain coronal sections (40 µm) which were collected in four parallel series. Sections were counterstained with Nissl staining to verify the placement of the electrodes.

### Data analysis

Raw signals were imported to the MATLAB development environment (The MathWorks, USA) for the off-line analysis using self-developed code.

As a first approach, field potentials components were quantified using the fast Fourier transform, revealing the power distribution in the frequency domain. Power spectra estimation was done by means of the Welch’s method (50% overlapping 4-s Hanning windows, with a fft size of 2.56 Hz and a nfft value of 1024). The frequency bands defined in the spectral analysis were: delta (0.5–4 Hz), theta (4–12 Hz), beta (12–30 Hz), gamma (30–40 Hz), low-gamma (40–70 Hz), and high-gamma (70–95 Hz).

#### Wavelet analysis

To reveal temporal changes in the oscillatory activity of the LFP time-series, the continuous wavelet transform was computed by using the MATLAB wavelet routines provided by C. Torrence and G. Compo^72^. Briefly, each signal was convoluted with the complex Morlet wavelet defined as:

1 $${{\rm{\Psi }}}_{0}(\eta )={\pi }^{-1/4}{e}^{i{\omega }_{0}\eta }{e}^{-{\eta }^{2}/2}$$

where ${\omega }_{0}$ is the wavelet central angle frequency. Here, ${w}_{0}=6$ is an optimal value for adjusting the time-frequency resolution^73^. The continuous wavelet transform of the sampled time series ${x}_{t}$ is the result of the convolution of the signal with the wavelet function, with a scaled and translated version of the parent wavelet function ${\Psi }_{0}(\eta )$,

2 $$W(t,s)=\langle {x}_{t}\otimes {\rm{\Psi }}(s,n)\rangle $$

where $\Psi (s,n)$ are scaled and shifted versions of the parent wavelet,

3 $${{\rm{\Psi }}}_{s,n}(t)={s}^{-\frac{1}{2}}\,{\rm{\Psi }}(\frac{t-n}{s})$$

The power of the signal at each wavelet scale $s$ (frequency) was defined as the modulus of the wavelet coefficient, $|W(t,s){|}^{2}$. The power values were normalised to scale, ${s}^{-1}(|W(t,s){|}^{2})$, to avoid scale-dependent biased values^74^. Finally, the spectrograms in the time-frequency domain were extracted with the z-score normalised power spectra when appropriate.

The time-averaged wavelet spectrum was used as a measure of the background spectrum, where peaks in the local wavelet spectra could be tested^72^:

4 $${W}^{2}(s)=\frac{1}{N}\sum _{n=0}^{N-1}{|{W}_{n}(s)|}^{2}$$

Whether a peak in the wavelet power spectrum is significantly above the background spectrum, then it be assumed to be a feature with a percent confidence (95% confidence level).

#### Synchronization measures

For detecting synchrony in a precise frequency range between two signals we used the methods purposed by Lachaux *et al*.^75^. The procedure computes a measure of phase-locking between the components of ${x}_{t}$ and ${y}_{t}$ signals at frequency $f$ by means of the phase relationship between the two signals. The instantaneous phase difference between both signals, $\varphi (t)$, was derived from the wavelet cross-spectrum^76, 77^. The cross-wavelet spectrum for the time series ${x}_{t}$ and ${y}_{t}$ is,

5 $${W}_{t}^{xy}(s)={W}_{t}^{x}(s){W}_{t}^{y\ast }(s)$$

where * is the complex conjugate. Then, the cross-wavelet phases were extracted as $\varphi (t,s)=ta{n}^{-1}( {\mathcal R} \{{W}_{t}^{xy}(s)\}/$ $\Im \{{W}_{t}^{xy}(s)\})$, where ${\mathcal R}$ and $\Im$ are the real and imaginary parts, respectively. The phase-locking value was defined for an epoch of length $N$ as

6 $$PLV=\frac{1}{N}|\sum _{n=1}^{N}\exp (i{\varphi }_{n})|$$

Phase synchrony was delimited to $PLV=1$ for exact phase synchrony, i.e., ${\varphi }_{n}=const$, and $PLV=0$ for no phase synchrony. We used the *phase lag index* (PLI) as a complement to obtain robust estimates of phase synchronization, against the presence of volume conduction, discarding phase differences that center around 0 *mod* π^78^. The index is derived from the phase difference distribution and can be obtained as follows:

7 $$PLI=|sign[{\rm{\Delta }}\varphi ]|$$

where ${\rm{\Delta }}\varphi$ is the phase differences.

Phase-difference values were selected from the cross-wavelet coefficients for the time-frequency regions isolated from the dominant frequency range for each time. In a first approach, the distributions of the phase difference values were represented on circular histograms with the radial extent of the circle segments representing the probability of the given phase-angles range. The Rayleigh’s test for relative phase value uniformity was used to test uniform distribution (null hypothesis) or whether significant phase preference was present ($p < 0.05$).

#### Phase-amplitude coupling

Cross-frequency interactions between different frequencies of a signal were assessed by the modulation index (MI), purposed by Tort *et al*.^79^. In general, raw time-series is filtered in the two frequency ranges of interest. MI is a normalized measure that reflects how well the instantaneous amplitude of a faster oscillation (*amplitude modulated*) with frequency band ${f}_{a}$, is phase-locked to a underlying lower cycle (*phase modulating*) with frequency band ${f}_{p}$. First, the raw signal is filtered at the two frequency ranges under analysis, obtaining ${x}_{{f}_{p}}$ and ${x}_{{f}_{A}}$. The instantaneous phase and amplitude of both processed signals are extracted by the Hilbert transform. Thus, amplitude envelope of ${x}_{{f}_{A}}$(${A}_{{f}_{p}}(t)$) and the instantaneous phases of ${x}_{{f}_{p}}$ (${\varphi }_{{f}_{A}}(t)$) gives the amplitude of the ${f}_{A}$ oscillation at each phase of the ${f}_{p}$ waves. Next, the binned phases and the mean of the amplitudes over each bin, the normalized mean amplitude is calculated:

8 $$P(j)=\frac{{\langle {A}_{{f}_{A}}\rangle }_{{\varphi }_{{f}_{p}}}(j)}{{\sum _{k=1}^{N}\langle {A}_{{f}_{A}}\rangle }_{{\varphi }_{{f}_{p}}}(k)}$$

The MI value was determined to test the amplitude locking of frequencies of interest. Phase was converted to the range −2π and +2π, with wave trough as the 0 radians. In the case of phase-amplitude coupling between both filtered signals, the amplitude distribution over the phase bins is non-uniform. Following Tort’s calculation, MI is defined as a measure that quantifies the deviation from the uniform distribution with an adaptation of the Kullback-Leibler distance^80^. Modulation index assumes normalized values between 0 and 1. The MI was considered statistically significant whether its value was >2 SD the mean surrogate MI, constructed by 1000 random permutations of the amplitude distribution.

### Statistical Analysis

Statistical analyses were performed using SPSS Statistics v20 (IBM, USA). Statistical comparisons were made using parametric or non-parametric tests wherever appropriate. We first checked whether the data fulfilled the conditions of normality (Kolmogorov–Smirnov test; *p* < 0.05 to reject) and homocedasticity (Levene’s test; *p* < 0.05 to reject). For the chemoexploratory behaviour analysis the data was log-transformed (log[X]) and an ANOVA (statistic *F*) was performed. The Kruskal–Wallis test (statistic *K*) was used as nonparametric method for comparisons between independent samples with pairwise comparisons (Mann–Whitney test with Bonferroni correction) when needed. To compare two independent samples, the Mann–Whitney test (statistic *U*) was used, and for the related samples comparisons the Wilcoxon test (statistic *W*) was performed. The minimum significance level for all the tests was 0.05.

### Data availability

The datasets generated during and/or analysed during the current study are available from the corresponding author on reasonable request.

## Electronic supplementary material

Supplementary Figures
