# Supplementary material for: Synchronized Activity in The Main and Accessory Olfactory Bulbs and Vomeronasal Amygdala Elicited by Chemical Signals in Freely Behaving Mice
Source: Sci Rep. 2017 Aug 30;7:9924. doi: 10.1038/s41598-017-10089-4 (PMC5577179; doi:10.1038/s41598-017-10089-4)
Supplement: Supplementary file 1 — Supplementary Figures [file 41598_2017_10089_MOESM1_ESM.pdf]

# TITLE

## SYNCHRONIZED ACTIVITY IN THE MAIN AND ACCESSORY OLFACTORY BULBS AND VOMERONASAL AMYGDALA ELICITED BY CHEMICAL SIGNALS IN FREELY BEHAVING MICE

# AUTHORS:

Cecília Pardo-Bellver[1,2], Sergio Martínez-Bellver[2], Fernando Martínez-García[3], Enrique Lanuza[1]* and Vicent Teruel-Martí[2]*

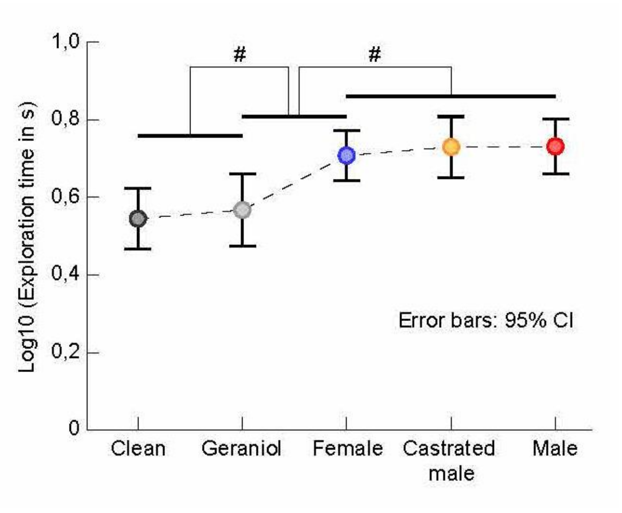


**Supplementary Figure 1**. Differences in the time spent exploring the presented stimuli. The differences in the means revealed three homogeneous subsets: neutral stimuli (clean and geraniol-scented bedding), conspecific-derived stimuli (female, castrated male and male-soiled bedding) and a mixed group (geraniol-scented bedding and female-soiled bedding).

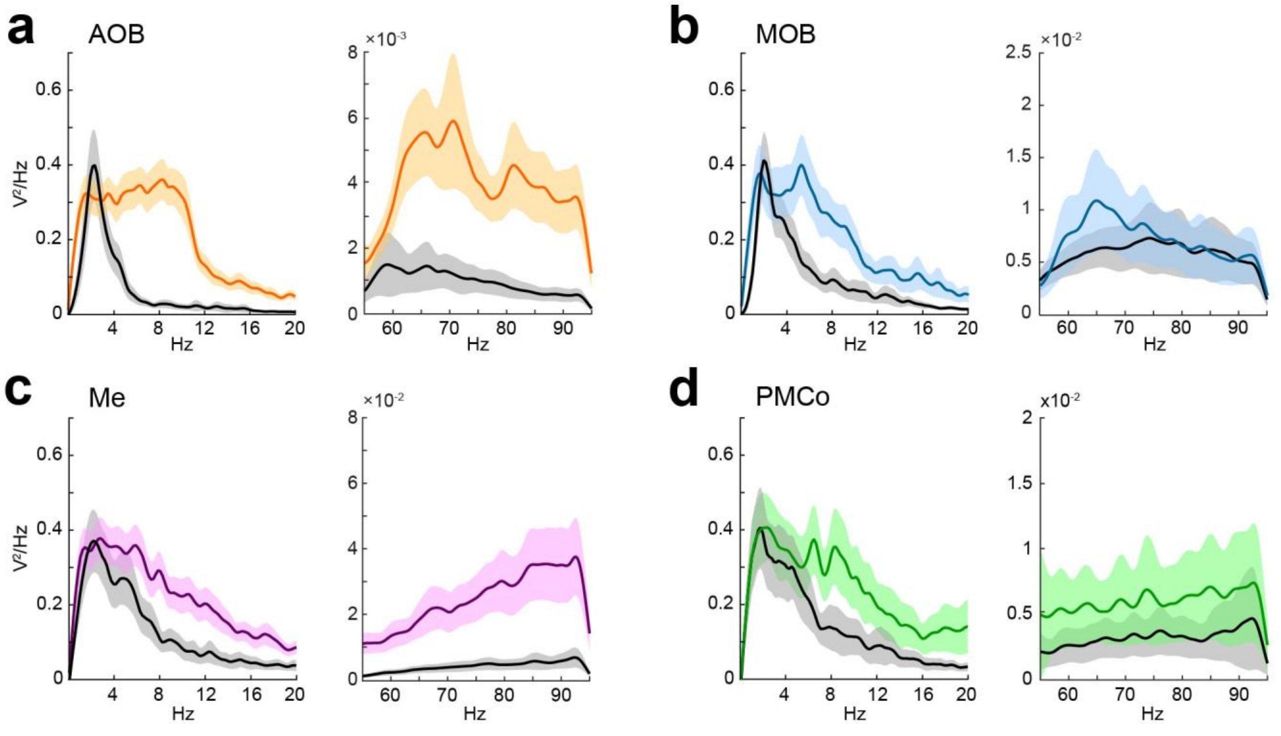


**Supplementary Figure 2**. Conspecific-odour-induced changes in the recorded nuclei. **a**, Power spectra in the AOB for male bedding (in colour) and non-exploration (in black), showing the average (solid line) and standard deviation (shadowed area) for the 0-20 Hz frequency band (left) and 55-95 Hz frequency band (right). Power spectra for male bedding in the MOB (**b**) and the Me (**c**); and for female bedding in the PMCo (**d**).

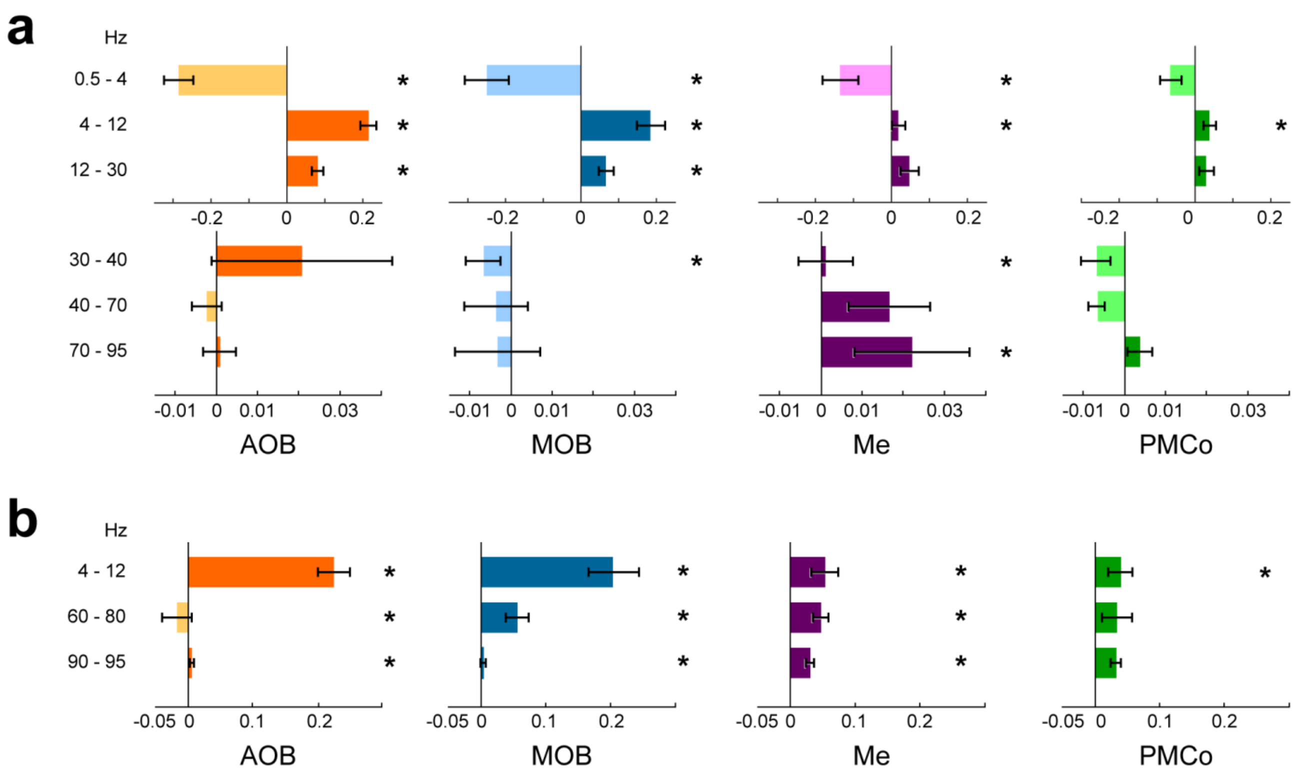


**Supplementary Figure 3.** Difference of the means of the band power ratio between the exploration (all stimuli) and the non-exploration conditions for different frequency bands. **a,** analysed as the band power ratio within the 0.5 – 95 Hz range. **b,** the theta (4 – 12 Hz) band power ratio was calculated within the 0.5 – 30 Hz range. According to the power spectra showed in Figure 5, the gamma intervals 60 – 80 Hz and 90 – 95 Hz were selected for a particular analysis, calculated within the 30 – 95Hz range.

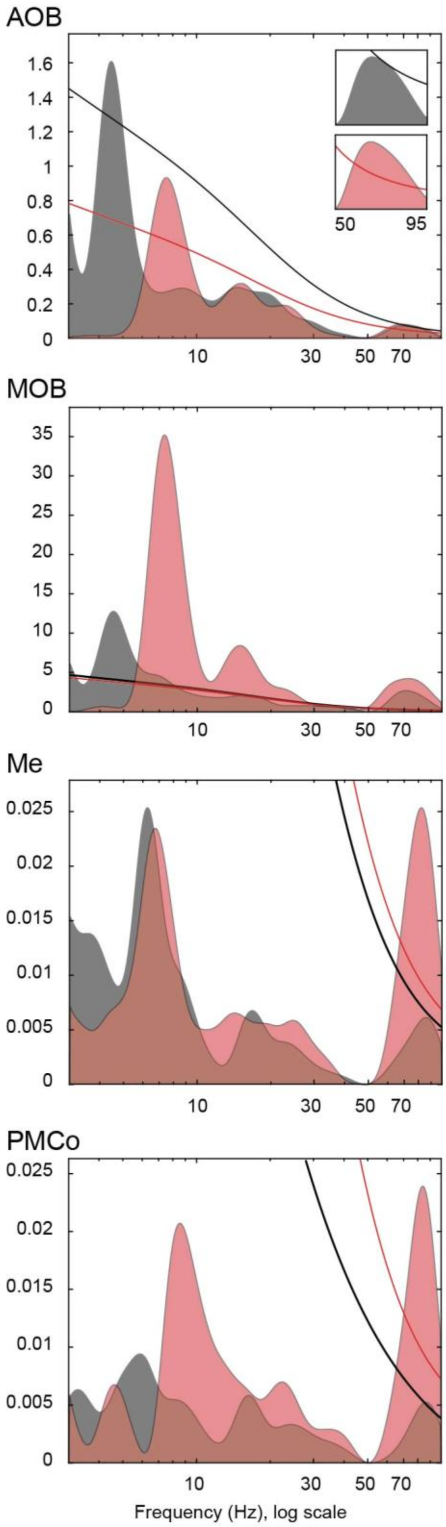


**Supplementary Figure 4**. Simultaneous gamma oscillations through the recorded nuclei. Time-averaged wavelet spectrum showing the comparisons between the peaks of power for a non-exploration period (in black) and an exploration period (in red). In the spectral distributions, gamma oscillations increase their powers above significant levels when engaged in exploration.

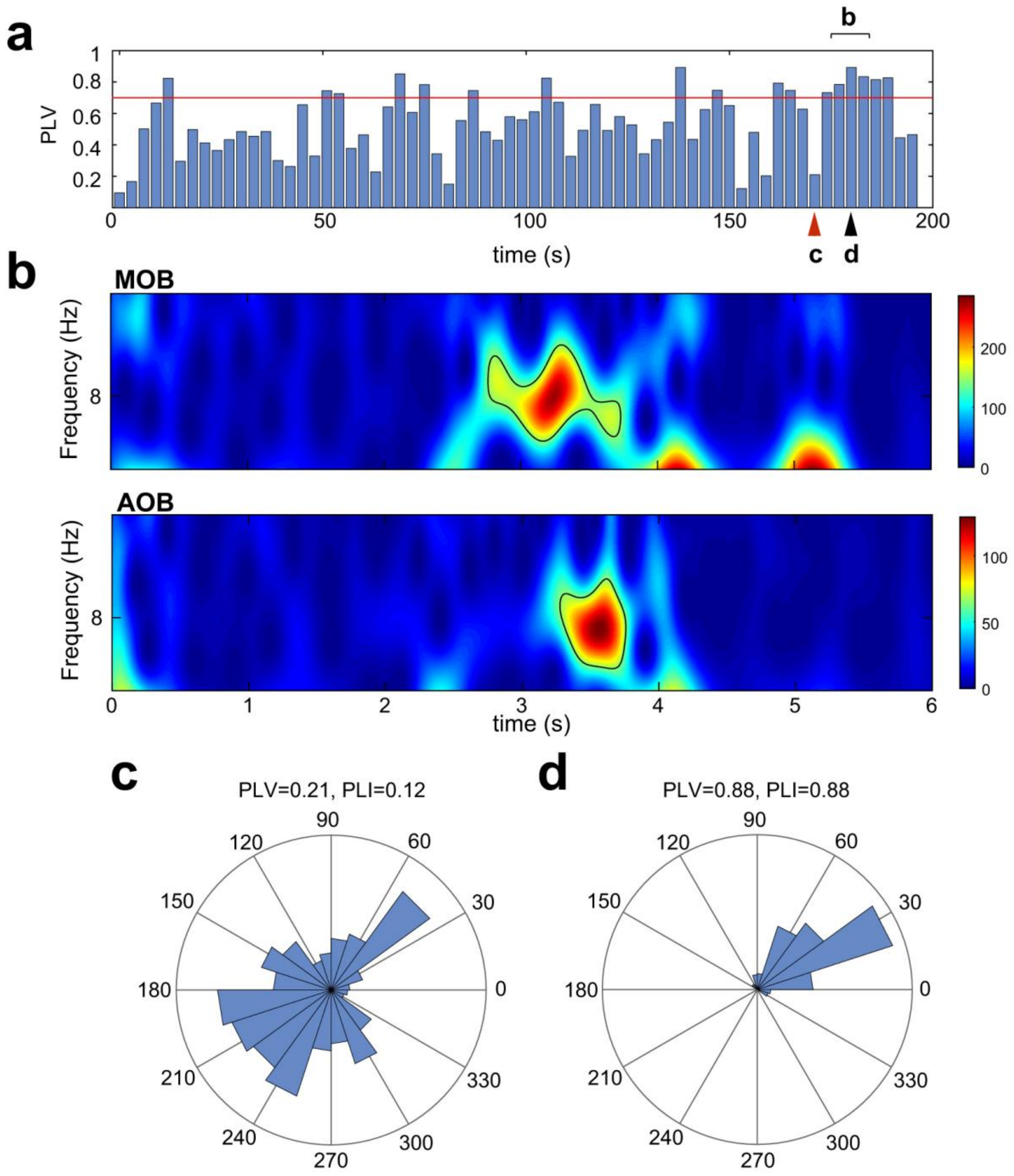


**Supplementary Figure 5**. Phase to phase synchronization between the AOB and the MOB for the clean bedding; the PLV was calculated filtering the signal at the main theta peak frequency (1 Hz wide). **a**. Time course of the PLV in a period of 200 seconds, including non-exploration, exploration and sniffing-like periods. Red line indicates the PLV 0.7 level, as threshold of significant coupling. b, Wavelet spectrogram of a representative epoch of sniffing-like event (marked in **a**). Powers included in the fourth percentile are delimited by a black isoline. **c**, circular diagram showing a representative case for a non-exploration time (marked in **a**), indicating the corresponding PLV and PLI values. **d**, idem for a sniffing-like period.

**Supplementary Table 1.** Results of the comparison of the power in the different frequency bands for exploration (all stimuli) and non-exploration/control times. * Indicates significant differences. Upper rows correspond to the analysis in the wide frequency range (0.5 – 95 Hz). Lower rows correspond to the analysis of the theta (relative to 0.5 – 30 Hz power ratio) and gamma (relative to 30 – 95 Hz power ratio).

| Nucleus | | Frequency band | *W* | N | *p* value |
|---|---|---|---|---|---|
| **AOB** | 0.5 – 95 Hz | 0.5 – 4 | -2.524 | 8 | 0.012 * |
| | | 4 – 12 | 2.524 | 8 | 0.012 * |
| | | 12 – 30 | 2.524 | 8 | 0.012 * |
| | | 30 – 40 | 0.841 | 8 | 0.400 |
| | | 40 – 70 | -0.421 | 8 | 0.674 |
| | | 70 – 95 | -0.140 | 8 | 0.889 |
| | 0.5 – 30 Hz | 4 – 12 | 2.366 | 7 | 0.018 * |
| | 30 – 95 Hz | 60 – 80 | -0.280 | 7 | 0.779 |
| | | 90– 95 | 2.243 | 7 | 0.025 * |
| **MOB** | 0.5 – 95 Hz | 0.5 – 4 | -3.059 | 12 | 0.002 * |
| | | 4 – 12 | 2.981 | 12 | 0.003 * |
| | | 12 – 30 | 2.824 | 12 | 0.005 * |
| | | 30 – 40 | -1.883 | 12 | 0.060 |
| | | 40 – 70 | -0.533 | 9 | 0.594 |
| | | 70 – 95 | -0.533 | 9 | 0.594 |
| | 0.5 – 30 Hz | 4 – 12 | 3.059 | 12 | 0.002 * |
| | 30 – 95 Hz | 60 – 80 | 2.310 | 9 | 0.021* |
| | | 90– 95 | 0.296 | 9 | 0.767 |
| **Me** | 0.5 – 95 Hz | 0.5 – 4 | -2.383 | 8 | 0.017 * |
| | | 4 – 12 | -0.561 | 8 | 0.575 |
| | | 12 – 30 | 1.963 | 8 | 0.050 * |
| | | 30 – 40 | 0.421 | 8 | 0.674 |
| | | 40 – 70 | 1.823 | 8 | 0.068 |
| | | 70 – 95 | 2.524 | 8 | 0.012 * |
| | 0.5 – 30 Hz | 4 – 12 | 2.103 | 8 | 0.035 * |
| | 30 – 95 Hz | 60 – 80 | 2.197 | 7 | 0.028 * |
| | | 90– 95 | 2.197 | 7 | 0.028 * |
| **PMCo** | 0.5 – 95 Hz | 0.5 – 4 | -1.820 | 8 | 0.069 |
| | | 4 – 12 | 2.521 | 8 | 0.012 * |
| | | 12 – 30 | 1.540 | 8 | 0.123 |
| | | 30 – 40 | -1.680 | 8 | 0.093 |
| | | 40 – 70 | -1.540 | 5 | 0.123 |
| | | 70 – 95 | 0.674 | 5 | 0.500 |
| | 0.5 – 30 Hz | 4 – 12 | 2.240 | 8 | 0.025 * |
| | 30 – 95 Hz | 60 – 80 | 0.944 | 5 | 0.345 |
| | | 90– 95 | 1.753 | 5 | 0.080 |
